# A UPLC-Q-TOF/MS-Based Metabolomics Study on the Effect of *Corallodiscus flabellatus* (Craib) B. L. Burtt Extract on Alzheimer's Disease

**DOI:** 10.1155/2021/8868690

**Published:** 2021-05-28

**Authors:** Yang-Yang Wang, Ning Zhou, Yan-Po Si, Zhi-Yao Bai, Meng Li, Wei-Sheng Feng, Xiao-Ke Zheng

**Affiliations:** ^1^Henan University of Chinese Medicine, 156 Jinshui East Road, Zhengzhou 450046, China; ^2^The Engineering and Technology Center for Chinese Medicine Development of Henan Province, 156 Jinshui East Road, Zhengzhou 450046, China; ^3^Key Laboratory of Chinese Materia Medica Ministry of Education, Heilongjiang University of Chinese Medicine, Harbin 150040, Heilongjiang, China

## Abstract

A UPLC-Q-TOF/MS-based metabolomics study was carried out to explore the intervening mechanism of *Corallodiscus flabellatus* (Craib) B. L. Burtt (CF) extract on Alzheimer's disease (AD). The AD model group consisted of senescence-accelerated mouse prone 8 (SAMP8) mice, and the control group consisted of senescence-accelerated mouse resistant 1 (SAMR1) mice. UPLC-Q-TOF/MS detection, multivariate statistical analysis, and pathway enrichment were jointly performed to research the change in metabolite profiling in the urine of AD mice. The result suggested that the metabolite profiling of SAMP8 mice significantly changed at the sixth month compared with SAMR1 mice of the same age, and the principal component analysis (PCA) score scatter plots of the CF group closely resembled those of the control and positive drug (huperzine A, HA) group. A total of 28 metabolites were considered potential biomarkers associated with the metabolism of beta-alanine, glycine, serine, threonine, cysteine, methionine, arginine, proline, and purines in AD mice. Furthermore, the CF group was clustered with the control and positive group and was clearly separated from the model group in the heat map. In conclusion, significant anti-AD effects were firstly observed in mice after treatment with the CF extract, and the urinary metabolomics approach assisted with dissecting the underlying mechanism.

## 1. Introduction

Alzheimer's disease (AD) is a debilitating neurodegenerative disease that usually occurs in elderly people over 65 years old, with primary symptoms of severe cognitive dementia, memory loss, learning function decline, and impaired behavior [1, 2]. At present, the pathogenesis of AD is not clear, and some studies proposed that oxidative stress, energy supply, and neuroinflammation in the brain play a prominent role in the initiation and development of dementia [3, 4]. Although there are many treatment strategies, there is no thoroughly effective therapy for AD due to various factors, including heredity, lifestyle, and environment [5]. However, traditional Chinese medicine (TCM) exhibits great potential for the treatment of disease [6]. Thus, there is great potential that a curative effect can be found for AD using Chinese herbal medicines or compounds.

*Corallodiscus flabellatus* (Craib) B. L. Burtt (CF) (family Gesneriaceae) is an herb that mainly grows in the provinces of Henan, Guangxi, and Yunnan in China. The entire plant is commonly used for treating upper respiratory tract infection, irregular menstruation, empyesis, and trauma [7]. Our previous work demonstrated that phenylethanoid glycosides and flavonoid glycosides are the main ingredients in CF [8–10]. They exhibited a variety of pharmacological activities, such as antioxygenation [11], anti-inflammation [12], antidementia, increased learning ability [13], and neuroprotection [14].

Based on high-throughput scale detection, a metabolomics study can reveal changes in the body due to environmental changes, drug effects, and endogenous stimulation. As an important part of systems biology, metabolomics can provide a comprehensive system-level method for studying the internal relationship between metabolites, disease, and drugs [15], and it is a powerful tool to study the mechanism of TCM.

In this study, an ultrahigh performance liquid chromatography-quadrupole time-of-flight-mass spectrometry (UPLC-Q-TOF/MS) based metabolomics approach was first applied to assess the changes in the metabolic network in AD model mice and in mice after they were treated with the CF extract. Then, a pattern recognition method combined with metabolic network analysis was applied to reveal the intervening mechanisms of CF on AD.

## 2. Materials and Methods

### 2.1. Materials

After its flowers bloom in September, *Corallodiscus flabellatus* B. L. Burtt was collected from the Funiu Mountain area of Xixia County, Henan Province, and identified by Professor Suiqing Chen of the Henan University of Chinese Medicine; voucher specimens (No. 20171025A) are kept in the Pharmaceutical Chemistry Laboratory of the Henan University of Chinese Medicine. CF extract was prepared as follows: 1 kg of dried CF was mixed with 12 times the amount of 50% ethanol (v/v) and was then heated to boiling for three continuous reflux extractions, 1 h each time. The liquid was filtered, and the filtrates from the three extractions were combined. The mixture was concentrated under reduced pressure and freeze-dried to obtain a total CF extract of 155 g, and the extraction rate was 15.5%. Huperzine A (HA) tablets were purchased from Henan Tailong Pharmaceutical Co. (Zhengzhou, China), production batch number 170103.

### 2.2. Reagents

Methanol and acetonitrile (HPLC grade) were purchased from Fisher Scientific (Bridgewater, NJ, USA). Deionized water was produced by the Molecular Water Purification system. Formic acid (LC/MS grade) was purchased from Fisher Scientific (Bridgewater, NJ, USA). Adenosine was purchased from Yuanye Bio-Technology Co., Ltd (Shanghai, China), production batch number Z23S7J21814.

### 2.3. Animals

The SPF male 6-month-old rapid-aging SAMP8 (as the model and medication groups, respectively) and anti-rapid-aging SAMR1 mice (as the control group) (weighing 29 ± 2 g) were purchased from the First Teaching Hospital of Tianjin University of TCM, license number: SCXK (Tianjin) 2014-0011. Mice were housed ten per cage under controlled conditions, at temperature 25 ± 2°C, relative humidity 40–70%, and 12 : 12 light/dark cycles with free access to standard food and water. All experiments were performed according to the regulations of the Experimental Animal Administration issued by the State Committee of Science and Technology of the People's Republic of China.

After 7 days of acclimatization, SAMR1 mice were set as the control group. SAMP8 mice were randomly split into 3 groups (10 per group): a model group, a positive drug group (huperzine HA, 0.02 mg/kg), and a CF group (775 mg/kg). The control and model group were treated with the same volume of distilled water, and all animals received intragastrical administration of the appropriate treatment one time each day for four consecutive weeks.

### 2.4. Urine Collection and Pretreatment

After four weeks of continuous treatment, at 12 hours after the last oral administration, all animals were housed in metabolic cages with 1 mouse per cage and drinking water ad libitum but without food for 12 h. All urine samples were immediately centrifuged at 3000 rpm for 10 min at 4°C to remove particulate matter after collection. Then, the supernatants were separated and stored at −80°C until UPLC-Q-TOF/MS analysis.

Urine samples were thawed at room temperature, and then each 300-*μ*l urine sample was mixed with 900 *μ*l ice-cold acetonitrile to precipitate protein. The mixture was vortexed for 3 min and centrifuged at 13,000 rpm for 10 min at 4°C. Finally, 2 *μ*l of the supernatant was injected into the UPLC system.

In addition, the quality control (QC) samples were mixed together the same volume from each group, respectively. The polled sample containing all analytes was used to provide a representative sample of average and used to assess the stability of the UPLC-MS system. In order to condition and equilibrate the system, the QC samples were injected five times at the beginning of the sequence, and the QC samples were also injected every six samples to further monitor the stability of the analytical result and evaluation the reliability of the metabolite profiling data [16].

### 2.5. Spectrum Acquisition

Spectrum acquisition was performed by an ultrahigh performance liquid chromatography system (Dionex UltiMate 3000 System, Thermo Scientific, USA) and screened with electrospray ionization-mass spectrometry (ESI-MS). The chromatographic separation was performed on an Acclaim TM RSLC 120 C18 column (2.2 *μ*m, 2.1 × 100 mm; Thermo Scientific, USA). The mobile phase consisted of solvent A (0.1% formic acid in water) and solvent B (acetonitrile) with a gradient elution (0-1 min, 98–84% A; 1–15 min, 84–81% A; 15–17 min, 81–2% A; 17–20 min, 2–2% A). The column was set at 40°C with a flow rate of 0.3 ml/min, and the temperature of the sample manager was set at 4°C.

Mass spectrometry analysis was performed using quadrupole time of flight mass spectrometry (Q-TOF/MS; maXis HD, Bruker, Germany) with an ESI source. Full scans were applied to both positive and negative modes. The mass data were acquired in centroid storage mode using a 50 to 1500 *m*/*z* scan range with a 1.0 Hz scan rate over the entire analysis. The following parameters were employed: the pressure of nebulizer gas, 2.0 Bar; drying gas (N_2_) flow rate, 8 L/min; drying gas temperature, 350°C; capillary voltage, 3.5 kV and 3.2 kV (in positive and negative mode, respectively).

### 2.6. Data Processing and Statistical Analysis

The raw mass data were calibrated, background noise was subtracted, and the peaks were aligned within Profile Analysis (version 2.1, Bruker, Germany), SIMCA-P (13.0, U metrics AB, Sweden) software. Then, the data were ready to be used for multivariate statistical analysis, which included principal component analysis (PCA) and orthogonal partial least square-discriminant analysis (OPLS-DA) combined with analysis of variance (ANOVA) to calculate the fold change and statistical significance to identify potential biomarkers. The biomarkers were filtered by the variable importance for the projection (VIP) values and *t* test (*P* < 0.05). In the PCA or OPLS-DA scores plot, *R*^2^ and *Q*^2^ play important roles in the quality of the fitting model, and they indicate the quality fitting of the variance in the model and the model's predictability of the variance in the raw data, respectively [17]. The metabolite peaks were assigned by the exact molecular weight and MS/MS information (see supplementary materials), combined with available biochemical databases, such as the Human Metabolome Database (HMDB) (http://www.hmdb.ca/), KEGG (http://www.genome.jp/kegg/), and METLIN (https://metlin.scripps.edu/).

MetPA (http://metpa.metabolomics.ca./MetPA/faces/Home.jsp) was used to analyze and visualize the pathway of potent biomarkers. It was also used to illustrate the biological context of the metabolic pathway, combining several advanced pathway enrichment analysis procedures along with the analysis of pathway topological characteristics to assist with the identification of the most relevant metabolic pathways involved in a given metabolomic study. Additionally, the heat map was created with Multiple Experiment Viewer (MeV) software (version 4.9.0).

## 3. Results

### 3.1. Method Validation

Considering the number of metabolites and the majority of molecular ions in the mass spectrum, quantitative information was obtained from a quality control (QC) sample. Seven ions (1.0 min, *m*/*z* 132.0768; 1.2 min, *m*/*z* 133.0608; 1.0 min, *m*/*z* 150.0585; 11.2 min, *m*/*z* 228.0630; 1.2 min, *m*/*z* 268.1038; 1.0 min, *m*/*z* 124.9914; 2.4 min, and *m*/*z* 144.0666) in the positive mode were selected to evaluate the reproducibility and stability of the adopted method. The relative standard deviations (RSDs) of peak areas and retention times for the selected ions were all less than 3.0% (Table 1). Consequently, the instrument precision and sample preparation procedure were highly acceptable for metabolomics analysis.

### 3.2. Metabolic Profiling Analysis

**Figure 1:**
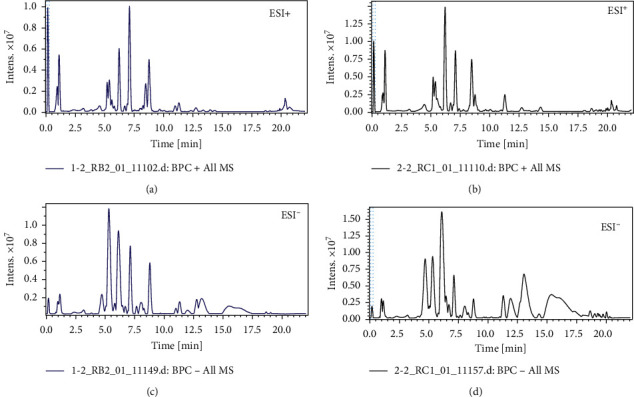
The typical BPCs of representative urine samples from (a, c) control mice and (b, d) AD mice in positive and negative modes.

**Figure 2:**
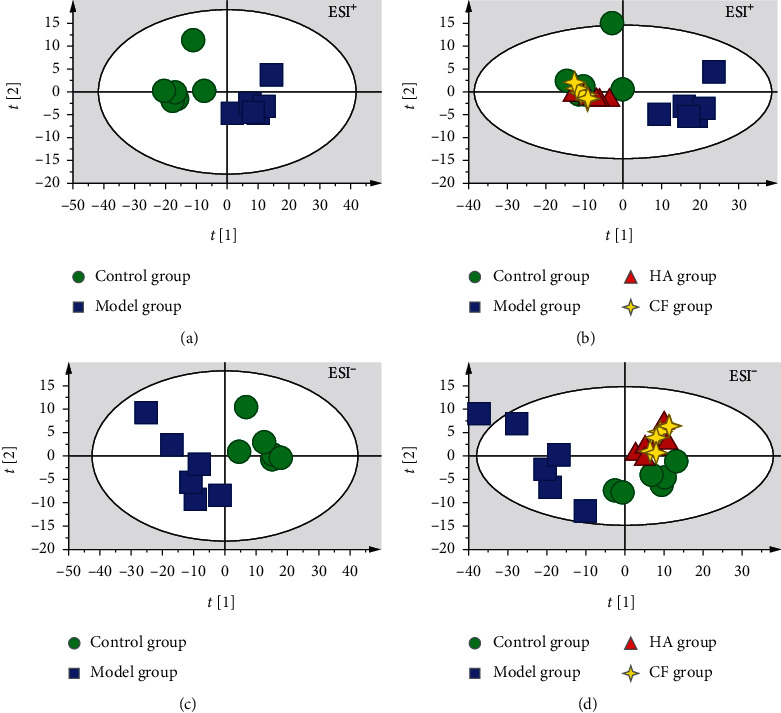
The PCA score plots of urine samples in positive and negative modes: (a) *R*^2^*X* = 0.942, *Q*^2^ = 0.788; (b) *R*^2^*X* = 0.928, *Q*^2^ = 0.797; (c) *R*^2^*X* = 0.91, *Q*^2^ = 0.718; (d) *R*^2^*X* = 0.918, *Q*^2^ = 0.716.

Using the optimal liquid chromatography-mass spectrometry (LC-MS) conditions described in Part 2.5, the representative basic peak chromatograms (BPC) of urine samples in positive and negative mode are shown in Figures 1(a)–1(d). Principal component analysis (PCA), a nonsupervised multivariate analytical method, was performed to visualize grouping trends among the control, AD model, and positive (huperzine A, HA) and CF treatment groups. There was a clear separation between the control and model groups in PCA score plots (Figures 2(a) and 2(c)), indicating that they had different metabolic profiles. Additionally, the CF treatment groups were gathered together with the control and positive groups (Figures 2(b) and 2(d)), indicating that the metabolic profile significantly changed in AD mice, and the CF extract revealed its therapeutic effects on AD.

### 3.3. Potential Biomarker Identification

**Figure 3:**
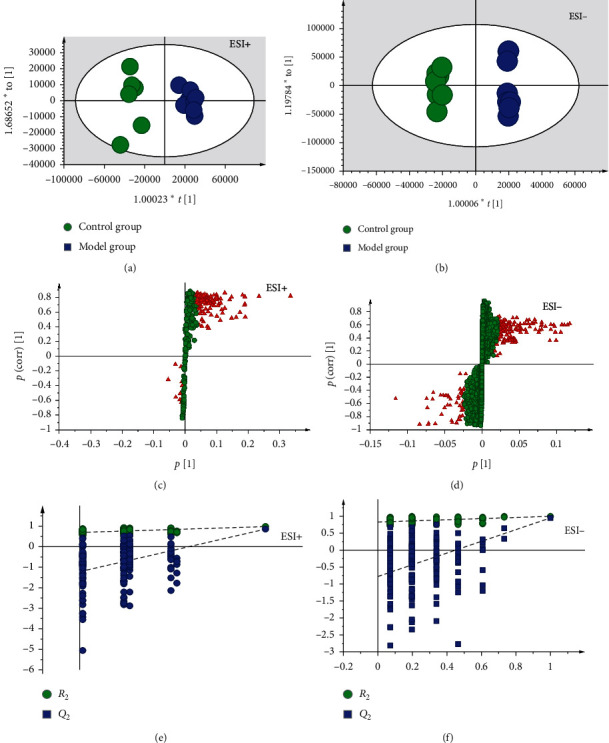
(a, b) The score plot and (c, d) S-plot of OPLS-DA obtained from the control and model group. (a) OPLS-DA score plot in positive mode *R*^2^*X* = 0.375, *R*^2^*Y* = 0.936, *Q*^2^ = 0.579; (b) OPLS-DA score plot in negative mode, *R*^2^*X* = 0.431, *R*^2^*Y* = 0.908, *Q*^2^ = 0.617; (c) S-plot score plot in positive mode, *R*^2^*X* = 0.684, *R*^2^*Y* = 0.991, *Q*^2^ = 0.843; and (e) S-plot score plot in negative mode, R^2^X = 0.569, R^2^Y = 0.831, *Q*^2^ = 0.735. (e, f) A 200-iteration permutation test showing that the values of permuted *R*^2^ and *Q*^2^ (bottom left) were significantly lower than the corresponding original *R*^2^ and *Q*^2^ values (top right), indicating that this OPLS-DA model was not overfitted.

The S-plot of the orthogonal partial least square-discriminant analysis (OPLS-DA) was used to construct a model to screen significantly changed metabolites in the AD group (Figure 3). A 200-iteration permutation test was used to assess the nonrandomness in positive and negative modes, and the results (Figures 3(e) and 3(f)) suggest that the quality of the OPLS-DA model was not overfitted and valid. Those metabolites were identified using the information of accurate mass, tandem mass spectrometry (MS/MS) fragments, authentic standards, and the origin in the Kyoto Encyclopedia of Genes and Genomes (KEGG) or Human Metabolome Database (HMDB) (supplementary materials). Then, the significantly changed metabolites were screened by VIP values (VIP > 3.0) and *t* test (*P* < 0.05).

Finally, a total of 28 potential biomarkers (19 in ESI^+^, and 9 in ESI^−^ mode) were identified and are listed in Table 2. Compared with the control group, 21 biomarkers were upregulated and 7 biomarkers downregulated in the AD model group. Moreover, nearly all biomarkers showed a significant reversal after CF extract administration.

### 3.4. Potential Biomarker Cluster Analysis

**Figure 4:**
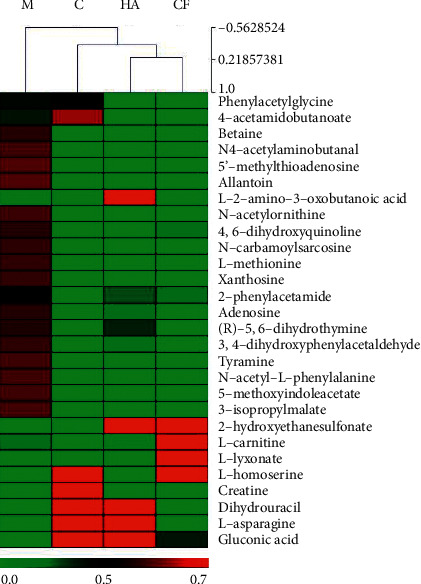
Heat map analysis of the potential biomarkers among the control group (C), model group (M), positive group (HA), and CF group. The *x*-axis represents the different groups, and the *y*-axis represents the different metabolites.

To present the level of global changes of differential metabolites, a heat map was generated to show the relative level of metabolites in each group (Figure 4). The results indicate that the CF group was clustered with the control and positive group and clearly separated from the model group, confirming the treatment effects of CF extract on AD mice.

### 3.5. Metabolic Pathway Analysis

**Figure 5:**
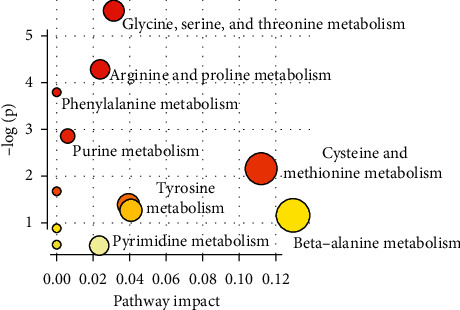
Metabolic enrichment analysis of biomarkers.

**Figure 6:**
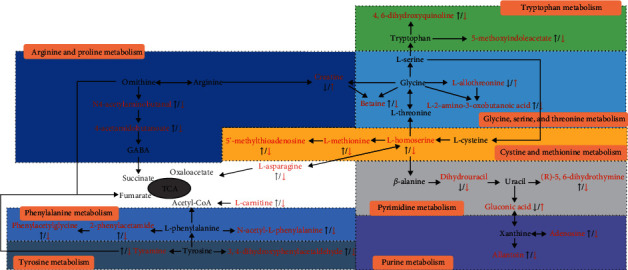
Metabolic network of differentiating metabolites in AD model mice.

In order to explore and visualize the various pathways affected by CF treatment, all the endogenous metabolites in Table 2 were imported into the MetaboAnalyst program. As shown in Figure 5, there are mainly three enriched metabolic pathways, including glycine, serine, and threonine metabolism, arginine and proline metabolism, and phenylalanine metabolism. Furthermore, MetPA was a useful tool for the visualization and integrative analysis of related metabolic pathways [18]. As a result, the disturbed metabolic network in AD mice was constructed as shown in Figure 6.

## 4. Discussion

Clinically, most diseases' diagnosis and prognosis detection was based on biological fluid samples, such as plasma, serum, and urine. This was mainly due to biological fluid easy to collect and directly reflect the overall state of the individual. Besides, it could monitor the biological response when the drug works in the body and provide additional information related to the organism state. The serum and plasma sample represents a “transient” state about the whole organism collecting the sample, while the urine sample represents an “average” state information as metabolism and excretion process in the body. So, in order to detect the effect of the drug on the organism's overall metabolic state, the urine sample was analyzed by metabolomic method to explore the mechanism of the intervention or treatment of CF on the AD mice.

### 4.1. Oxidation Injury and the Effect of CF Extract on AD

#### 4.1.1. Cysteine and Methionine Metabolism

In cysteine and methionine metabolism, as Table 2 shows, 2-hydroxyethanesulfonate decreased in AD model mice and increased after the CF extract was administered to the AD mice. 2-hydroxyethanesulfonate and taurine can transform into each other under the effect of ferricytochrome-coxidoreductase and sulfoacetaldehyde reductase. The 2-hydroxyethanesulfonate level was decreased in the urine of the AD model group, suggesting that the taurine may have decreased as well. Taurine acts as an antioxidant, neuromodulator [19], and cytomembrane stabilizer in the brain [20], which plays a key role in the central nervous system. Therefore, the effect of CF extract on AD mice could thoroughly decrease the level of homocysteine and inhibit the oxidation of the nervous system in the brain.

In addition, l-methionine is the essential sulfur-containing amino acid that participates in cysteine and methionine metabolism. Homocysteine is known to be a precursor for l-methionine, which can be transformed into homocysteine by a related pathway. In this study, the level of l-methionine in the model group increased, indicating that the level of homocysteine will subsequently be raised. Additionally, an elevated level of homocysteine in cerebrospinal fluid (CSF) and blood plasma could accelerate the accumulation of *β*-amyloid protein in the brain and increase the risk of AD [21, 22]. In the current study, it was observed that the level of l-methionine decreased in the CF extract group, showing the curative effect on AD mice.

#### 4.1.2. Tryptophan Metabolism

In the tryptophan metabolism pathway, 4,6-dihydroxyquinoline and 5-methoxyindoleacetate were significantly increased. This indicated that the tryptophan metabolism pathway was remarkably disturbed in the AD model mice [23, 24] and the level of tryptophan may increase as well. Under the effect of related enzymes, a series of metabolites including serotonin, melatonin, niacin, and quinolinic acid can be produced by tryptophan catabolism [25], which plays a key role in the pathobiology of AD at an early stage [26]. In addition, quinolinic acid acts as a chronic inducer and immunomodulator to generate *β*-amyloid polypeptide and reactive oxygen species (ROS) [27]. However, the CF extract could significantly decrease those metabolite levels and further decrease *β*-amyloid polypeptide deposited and oxidized by tryptophan metabolism.

#### 4.1.3. Purine Metabolism

In the purine metabolism pathway, allantoin is excreted as a final product in the body in a reaction where various ROS are oxidized. The level of allantoin could increase in AD [28] and could occur prior to the beginning of AD symptoms [29]. As one of the important mechanisms, injury of brain tissue caused by oxidized proteins could promote AD occurrence [30]. Furthermore, the level of allantoin in ischemia-reperfusion injury [31] and atherosclerosis [32] models were raised, which was closely related to oxidative stress. In this study, CF extract downgraded allantoin levels by purine metabolism and further increased the oxidation in the brain.

### 4.2. Energy Metabolism Failure and the Effect of CF Extract on AD

#### 4.2.1. Arginine and Proline Metabolism

Creatine is a key metabolite in arginine and proline metabolism. Creatine participates in buffering intracellular energy stores by activation of the creatine kinase/phosphocreatine system that plays an integral role in energy buffering and cellular bioenergetics [33]. It was observed that the level of creatine was decreased in the CSF, hippocampus, and plasma of AD patients [34–36], and the degenerative state of the central nervous system could be attenuated after supplementation with creatine [34, 37]. As the result shows in Table 2, the level of creatine in the urine of AD model mice was significantly lower than that in the control mice, indicating that the CF extract could increase the energy supplementation in the brain by increasing creatine levels.

#### 4.2.2. Alanine, Aspartate, and Glutamate Metabolism

In the alanine, aspartate, and glutamate metabolism pathway, *N*-acetylaspartate (NAA) is seen as a potential biomarker of AD in early diagnoses. Lower NAA levels are strongly associated with mitochondrial dysfunction and energy deficiency in the brain, and decreased NAA is one of the key factors associated with neuronal cell death [38–41]. In addition, a decrease in NAA is commonly associated with neurotoxicity, neuronal loss, and neurodegeneration in AD [42, 43]. In this study, the level of NAA decreased in AD model mice, and CF extract increased NAA levels. Thus, it was shown that the situation could be improved in AD mice after treating with CF extract to increase NAA levels.

### 4.3. Neuromodulator Disorder in AD and the Effect of CF Extract on AD

#### 4.3.1. Purine Metabolism

Xanthosine and adenosine are major metabolites of purine metabolism. In purine metabolism, xanthosine is converted to xanthine under the nucleoside phosphorylase affected, and disorders of xanthine levels might point to their importance in the process of tangle formation in AD [44]. As one factor of neurodegenerative disease [45], the xanthine concentration increased in the hippocampus of an AD patient, which may have occurred due to the loss of the A1 receptor [46]. In the current study, the decreased level of xanthosine in the CF extract group compared with the AD model group indicated that CF extract might inhibit xanthine levels and relieve the process of tangle formation.

Adenosine is an important neuromodulator that plays a significant role in nervous system homeostasis with the A1, A2a, A2b, and A3 receptors [47, 48]. Adenosine receptors, as G-protein-coupled receptors, can regulate the second messenger cAMP in opposite directions and regulate both synaptic transmission and plasticity. When the expression of adenosine receptors A1 and A2a is imbalanced, this could lead to dysfunction of cognition [49]. CF extract and huperzine A were observed to prevent an increase in adenosine in AD model mice, indicating that the effects of CF extract may be based on the regulation of the dysfunction of adenosine receptors by purine metabolism.

#### 4.3.2. Arginine and Proline Metabolism

*γ*-Aminobutyric acid (GABA) is an inhibitory amino acid that can reduce the release of glutamic acid and antagonize glutamate toxicity to protect nerve cells [50]. Previous reports indicated that increasing and maintaining the level of GABA for neurons in the brain could efficiently improve and repair related learning and memory impairment [51–54]. Conversely, a decrease in the level of GABA could lead to memory impairment in AD patients. Interesting, N4-acetylaminobutanal and 4-acetamidobutanoate are two adjacent metabolites in the arginine and proline metabolism pathway. N4-acetylaminobutanal is transformed into 4-acetamidobutanoate in the metabolic process under NAD+, and to *γ*-aminobutyric acid (GABA) under deacetylase. In AD model mice, the levels of the GABA precursors N4-acetylaminobutanal and 4-acetamidobutanoate were increased, which may be due to the low activity of the enzyme deacetylase, which leads to the accumulation of the precursors and limited the generates of GABA. However, CF extract can restore those metabolite levels by alanine, aspartate, and glutamate metabolism.

#### 4.3.3. Glycine, Serine, and Threonine Metabolism

L-carnitine is a derivative of L-lysine, and it acts as a long-chain fatty acid transporter in the *γ*-oxidation cycle and neutralizes toxic acyl-CoA in mitochondria [55, 56]. Because it is involved in cholinergic neurotransmission, its important property is neuroprotective, and it neutralizes the brain damage induced by oxidative stress [57]. In our study, the level of L-carnitine was increased in the urine of AD model mice, which could be due to increasing biomarkers of oxidative stress after AD occurs [58] or the different effects of serum and urine in the body due to the compositional difference. Meanwhile, the lower level of L-carnitine showed the CF extract showed the ability of antioxidative.

The spatial learning and memory of AD model mice were improved, and symptoms were decreased in the CF-treated group. However, in this study, the metabolic profile and the effect of CF extract on AD were investigated by the method of metabolomics for the first time. This mainly occurred through arginine and proline metabolism; cysteine and methionine metabolism; purine metabolism; alanine, aspartate, and glutamate metabolism; tryptophan metabolism; glycine, serine, and threonine metabolism pathways to adjust the disordered metabolites in AD model mice. These disturbances could be rectified by CF extract, which corrected multiple pathways to ameliorate the AD symptoms.

## 5. Conclusions

Our prophase research has proved the efficacy of CF extract on AD mice from a pathological point of view and has also explored the molecular mechanism. Herein, a UPLC-QTOF-MS-based urine metabolomics study was performed to illustrate the metabolic characteristics of the AD model and the therapeutic effects of the CF extract. A total of 28 potential biomarkers related to AD were detected, and the metabolic pathways were established by MetPA. Pattern recognition with multivariate statistical analysis and clustering analysis with heat maps showed that the metabolic profile of the AD model group was clearly separated from the control group, and the CF group was closer to the control group. The metabolic pathways concerning oxidation, energy metabolism failure, and neuromodulator disorder were elucidated in our study. Moreover, this was the first metabolomics study to determine the therapeutic mechanism of the CF extract on AD model mice.

## Figures and Tables

**Table 1:** The relative standard deviations of area peaks and retention times for typical metabolites.

ESI mode	Metabolite	*m*/*z*	RSD (peak area) (%)	RSD (retention time) (%)
ESI^+^	Creatine	131.0695	1.50	0.49
	L-asparagine	132.0535	2.92	0.51
	L-methionine	149.0510	1.60	0.53
	5-methoxyindoleacetate	205.0739	1.41	0.11
	Adenosine	267.0968	2.22	0.42

ESI^−^	2-hydroxyethanesulfonate	125.9987	2.05	0.46
	Allantoin	158.0440	1.49	0.52

Note: ESI = electrospray ionization.

**Table 2:** The potential biomarkers and changing trends.

Mode	Number	RT (min)	Compound	Formula	Determined (*m/z*)	Ion form	Trends
ESI^+^	1	1.2	Dihydrouracil	C_4_H_6_N_2_O_2_	115.0502	[M + H]^+^	**↓** ^*∗*^ **↓**
	2	1	Betaine	C_5_H_11_NO_2_	118.0862	[M + H]^+^	**↑** ^∗∗^ **↓** ^**##**^
	3	1	L-allothreonine	C_4_H_9_NO_3_	120.0653	[M + H]^+^	**↓** ^*∗*^ **↑**
	4	1.2	(R)-5,6-dihydrothymine	C_5_H_8_N_2_O_2_	129.0656	[M + H]^+^	**↑** ^∗∗^ **↓** ^**##**^
	5	1	N4-acetylaminobutanal	C_6_H_11_NO_2_	130.0862	[M + H]^+^	**↑** ^∗∗^ **↓** ^**##**^
	6	1	Creatine	C_4_H_9_N_3_O_2_	132.0768	[M + H]^+^	**↓** ^*∗*^ **↑**
	7	1.2	L-asparagine	C_4_H_8_N_2_O_3_	133.0608	[M + H]^+^	**↓** ^*∗*^ **↑**
	8	6.8	N-carbamoylsarcosine	C_4_H_8_N_2_O_3_	133.1009	[M + H]^+^	**↑** ^∗∗^ **↓** ^**##**^
	9	1.2	Tyramine	C_8_H_11_NO	138.0908	[M + H]^+^	**↑** ^∗∗^ **↓** ^**##**^
	10	1	L-methionine	C_5_H_11_NO_2_S	150.0585	[M + H]^+^	**↑** ^∗∗^ **↓** ^**##**^
	11	6.2	4,6-dihydroxyquinoline	C_9_H_7_NO_2_	162.0549	[M + H]^+^	**↑** ^∗∗^ **↓** ^**##**^
	12	1	L-carnitine	C_7_H_15_NO_3_	162.1124	[M + H]^+^	**↑** ^∗∗^ **↓** ^**##**^
	13	6.6	N-acetylornithine	C_7_H_14_N_2_O_3_	175.1074	[M + H]^+^	**↑** ^∗∗^ **↓** ^**##**^
	14	9.8	N-acetyl-L-phenylalanine	C_11_H_13_NO_3_	208.0967	[M + H]^+^	**↑** ^∗∗^ **↓** ^**##**^
	15	7	Phenylacetylglycine	C_10_H_11_NO_3_	216.0626	[M + Na]^+^	**↑** ^∗∗^ **↓** ^**##**^
	16	11.2	5-methoxyindoleacetate	C_11_H_11_NO_3_	228.0630	[M + Na]^+^	**↑** ^∗∗^ **↓** ^**##**^
	17	1.2	Adenosine	C_10_H_13_N_5_O_4_	268.1038	[M+H] ^+^	**↑** ^∗∗^ **↓** ^**##**^
	18	6.6	Xanthosine	C_10_H_12_N_4_O_6_	285.0843	[M+H] ^+^	**↑** ^∗∗^ **↓** ^**##**^
	19	5.2	5'-methylthioadenosine	C_11_H_15_N_5_O_3_S	298.0965	[M+H] ^+^	**↑** ^∗∗^ **↓** ^**##**^

ESI^−^	20	1.2	L-2-amino-3-oxobutanoic acid	C_4_H_7_NO_3_	116.0354	[M-H]^−^	**↑ ↓**
	21	1.0	2-hydroxyethanesulfonate	C_2_H_6_O_4_S	124.9914	[M-H]^−^	**↓ ↑** ^**#**^
	22	6.2	2-phenylacetamide	C_8_H_9_NO	134.0610	[M-H]^−^	**↑** ^*∗*^ **↓**
	23	2.4	4-acetamidobutanoate	C_6_H_11_NO_3_	144.0666	[M-H]^−^	**↑ ↓** ^**#**^
	24	7.4	3,4-dihydroxyphenylacetaldehyde	C_8_H_8_O_3_	151.0398	[M-H]^−^	**↑** ^*∗*^ **↓**
	25	1.0	Allantoin	C_4_H_6_N_4_O_3_	157.0368	[M-H]^−^	**↑ ↓**
	26	1.2	L-lyxonate	C_5_H_10_O_6_	165.0404	[M-H]^−^	**↓ ↑** ^**#**^
	27	5.8	3-isopropylmalate	C_7_H_12_O_5_	175.0611	[M-H]^−^	**↑** ^∗∗^ **↓** ^**##**^
	28	1.0	Gluconic acid	C_6_H_12_O_7_	195.0511	[M-H]^−^	**↓** ^*∗*^ **↑**

^*∗*^ *p* < 0.05 and ^∗∗^*p* < 0.01 model group compared with the control group; #*p* < 0.05 and ##*p* < 0.01 CF group compared with the model group. **↓**/**↑**represent the trends in the model and CF groups, respectively.

## Data Availability

The data used to support the findings of this study are available from the corresponding author upon request.

## Supplementary Materials

Supplementary Materials See Figure S1 in the Supplementary Material for metabolites identified, which is based on standard and MS/MS combined with METLIN database.
