# Supplementary material for: A UPLC-Q-TOF/MS-Based Metabolomics Study on the Effect of Corallodiscus flabellatus (Craib) B. L. Burtt Extract on Alzheimer's Disease
Source: Evid Based Complement Alternat Med. 2021 May 28;2021:8868690. doi: 10.1155/2021/8868690 (PMC8177975; doi:10.1155/2021/8868690)

**Graphical abstract**

**UHPLC-Q-TOF/MS based metabolomics study of**

***Corollaodiscus flabellate* (Craib) B. L. Burtt extract on Alzheimer’s disease**

A UPLC-Q-TOF/MS based metabolomics study was carried out to explore the intervening mechanism of *Corollaodiscus flabellate* (Craib) B.L. Burtt (CF) extract on Alzheimer’s disease (AD). It’s revealed that CF extract exhibited significant anti-AD effects and the urinary metabolomics approach could dissect the underlying mechanism.


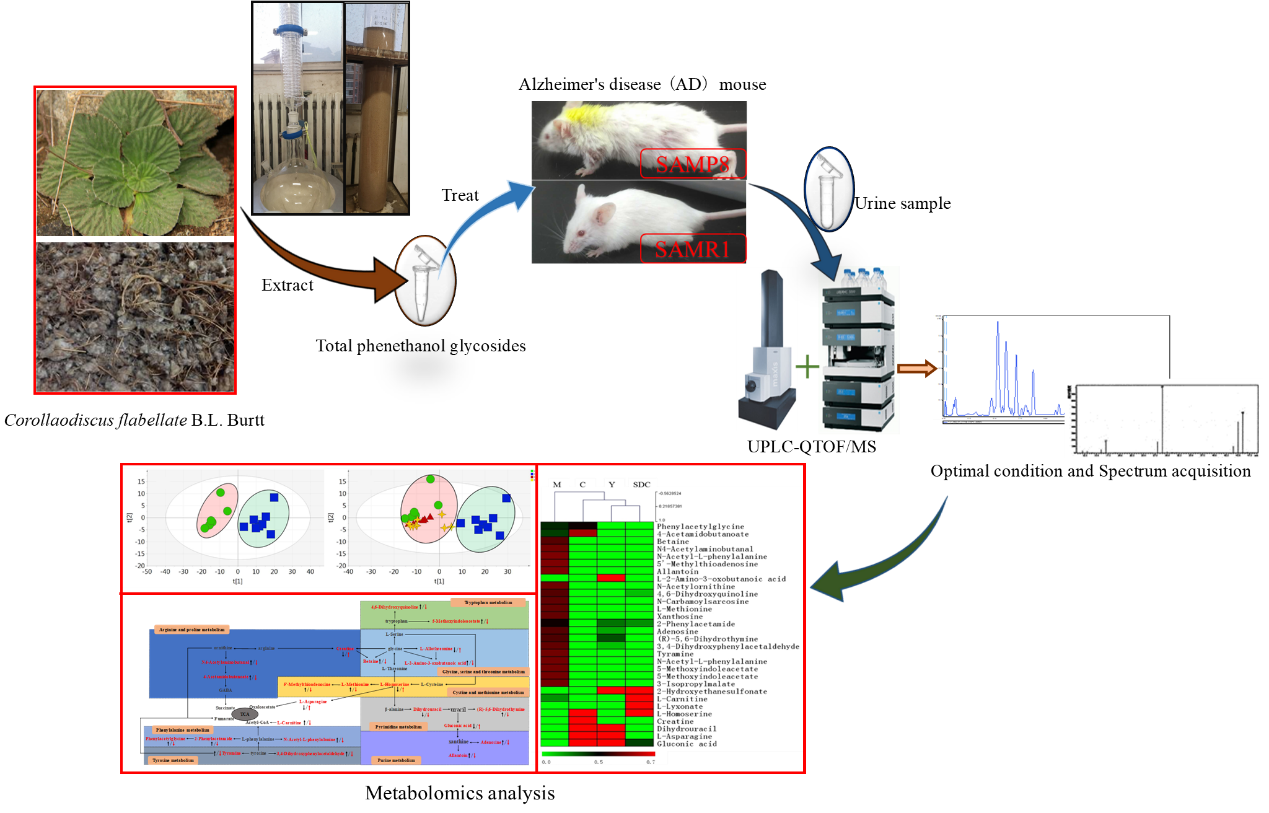

Supplement: Supplementary Materials — See Figure S1 in the Supplementary Material for metabolites identified, which is based on standard and MS/MS combined with METLIN database. [file 8868690.f1.docx]
